# Analysis of Copy Number Variants on Chromosome 21 in Down Syndrome-Associated Congenital Heart Defects

**DOI:** 10.1534/g3.117.300366

**Published:** 2017-11-15

**Authors:** Benjamin L. Rambo-Martin, Jennifer G. Mulle, David J. Cutler, Lora J. H. Bean, Tracie C. Rosser, Kenneth J. Dooley, Clifford Cua, George Capone, Cheryl L. Maslen, Roger H. Reeves, Stephanie L. Sherman, Michael E. Zwick

**Affiliations:** * Department of Human Genetics, Emory University School of Medicine, Atlanta, Georgia 30322; † Department of Epidemiology, Rollins School of Public Health, Emory University, Atlanta, Georgia 30322; ‡ Department of Pediatrics, Sibley Heart Center Cardiology, Children’s Healthcare of Atlanta, Atlanta, Georgia 30033; § Heart Center, Nationwide Children’s Hospital, Columbus, Ohio 43205; ** Kennedy Krieger Institute, Baltimore, Maryland 21205; †† Knight Cardiovascular Institute, Oregon Health & Science University, Portland, Oregon 97239; ‡‡ Department of Molecular and Medical Genetics, Oregon Health & Science University, Portland, Oregon 97239; §§ Department of Physiology, School of Medicine, Johns Hopkins University, Baltimore, Maryland 21205; *** McKusick Nathans Institute for Genetic Medicine, School of Medicine, Johns Hopkins University, Baltimore, Maryland 21205

**Keywords:** Down syndrome, congenital heart defects, copy number variation

## Abstract

One in five people with Down syndrome (DS) are born with an atrioventricular septal defect (AVSD), an incidence 2000 times higher than in the euploid population. The genetic loci that contribute to this risk are poorly understood. In this study, we tested two hypotheses: (1) individuals with DS carrying chromosome 21 copy number variants (CNVs) that interrupt exons may be protected from AVSD, because these CNVs return AVSD susceptibility loci back to disomy, and (2) individuals with DS carrying chromosome 21 genes spanned by microduplications are at greater risk for AVSD because these microduplications boost the dosage of AVSD susceptibility loci beyond a tolerable threshold. We tested 198 case individuals with DS+AVSD, and 211 control individuals with DS and a normal heart, using a custom microarray with dense probes tiled on chromosome 21 for array CGH (aCGH). We found that neither an individual chromosome 21 CNV nor any individual gene intersected by a CNV was associated with AVSD in DS. Burden analyses revealed that African American controls had more bases covered by rare deletions than did African American cases. Inversely, we found that Caucasian cases had more genes intersected by rare duplications than did Caucasian controls. We also showed that previously DS+AVSD (DS and a complete AVSD)-associated common CNVs on chromosome 21 failed to replicate. This research adds to the swell of evidence indicating that DS-associated AVSD is similarly heterogeneous, as is AVSD in the euploid population.

Understanding the rules by which variation that influences genome dosage also impacts phenotypes remains one of the central challenges of human genetics (Zarrei *et al.* 2015). DS, caused largely by trisomy 21, provides an extreme example of a dosage change that impacts many aspects of an individual’s phenotype. Congenital heart defects (CHDs) are among the most common and significant birth defects found in individuals with DS. In the disomic population, CHDs are the most common birth defect, presenting in 80 out of 1000 live births and causing 25% of infant mortality (Reller *et al.* 2008; Yang *et al.* 2002; Hartman *et al.* 2011; Mai *et al.* 2015). For children with trisomy 21, CHD incidence is substantially higher: nearly 450 out of 1000 live births have a CHD (Loffredo *et al.* 2001; Freeman *et al.* 2008).

AVSDs are a serious CHD resulting from the failure of endocardial cushion formation and subsequent mitral and tricuspid valve formation. In the presence of an AVSD, there is improper mixing of oxygenated and deoxygenated blood. While the heart is typically repaired during the first year of life, patients with AVSD face increased risk of sequelae, including arrhythmias, endocarditis, stroke, congestive heart failure, pulmonary hypertension, and continued heart valve problems (Le Gloan *et al.* 2011). Trisomy 21 is the single greatest risk factor for AVSD. While 1 in 10,000 people in the general population present with AVSD, among infants with DS, the rate is 1 in 5 (Freeman *et al.* 2008). This 2000-fold greater risk suggests that those with DS represent a sensitized population in which genetic variation contributing to the risk for AVSD may have a larger effect size than in the general population. Using the DS population to identify AVSD risk loci may therefore yield high statistical power, even with a small sample size (Zwick *et al.* 1999).

In our prior study, the largest genetic study of its kind to date, we characterized genome-wide CNVs in a well-phenotyped cohort with 210 case individuals with DS+AVSD and 242 control individuals with DS and structurally normal hearts (DS+NH) (Ramachandran *et al.* 2015a). We showed a statistically significant increase in large, rare deletions in DS+AVSD cases that also impacted more genes than those in DS+NH controls. Gene set enrichment tests suggested an enrichment of large deletions intersecting ciliome genes. Most importantly, the scale of this study showed that, even in the sensitized DS population, there are no large, common CNVs with a major effect on AVSD that could account for the 2000-fold increased risk in DS. We have also shown that common SNPs cannot account for the increased risk of CHD in this same cohort (Ramachandran *et al.* 2015).

In the current study, we focus specifically on CNVs on chromosome 21 and test two primary hypotheses: (1) individuals with DS carrying chromosome 21 deletions may be protected from AVSD, because these deletions return AVSD susceptibility loci back to disomy, and (2) individuals with DS carrying chromosome 21 duplications are at increased risk for AVSD, because these duplications boost the dosage of AVSD susceptibility loci beyond a tolerable threshold. In addition to testing these hypotheses, we used our large cohort of 198 DS+AVSD cases and 211 DS+NH controls in an independent replication of Sailani *et al.* (2013). They screened for CNVs on chromosome 21 in a similarly defined DS cohort of 55 DS+AVSD cases and 53 DS+NH controls and reported two common CNVs significantly associated with AVSD.

## Materials and Methods

### DNA samples

Participant samples were collected as described previously (Freeman *et al.* 1998, 2008; Locke *et al.* 2010; Ramachandran *et al.* 2015a,b). Individuals diagnosed with full or translocation trisomy 21, documented by karyotype, were recruited from centers across the United States. Institutional review boards at each enrolling institution approved protocols, and informed consent was obtained from a custodial parent for each participant. A single cardiologist (K. Dooley) identified cases from medical records as individuals with a complete, balanced AVSD diagnosed by echocardiogram or surgical reports (DS+AVSD). Controls were classified as individuals with a structurally normal heart, patent foramen ovale, or patent ductus arteriosus (DS+NH).

Genomic DNA was extracted from LCLs with the Puregene DNA purification kit according to the manufacturer’s protocol (QIAGEN, Valencia, CA). DNA quantity and quality were checked on a NanoDrop ND-1000 spectrophotometer (NanoDrop Technologies, Wilmington, DE) and assessed for integrity on 0.8% agarose gels stained with ethidium bromide.

### Microarray design and processing

All analyses used the human genome reference hg19 build. We designed a custom 8x60k Agilent (Agilent Technologies, Santa Clara, CA) CGH array using eArray (https://earray.chem.agilent.com accessed April 2011). The array consisted of 52,944 60-mer DNA probes targeting human chromosome 21, providing a mean spacing of 673 bp and a median spacing of 448 bp, as well as a genomic backbone of probes and Agilent’s control probes (design file: ADM2Chr21_60k_final_033839_D_F_20120731.xml).

Array hybridization was processed according to Agilent’s protocol and scanned on an Agilent SureScan High-Resolution Microarray Scanner at Emory University. A single female (GEO accession individual ID: 246) with trisomy 21 and no CHD was used as the reference sample for all test individuals. This individual had a known deletion at chr21:45,555,257–45,615,042, which would be detected as a duplication in all test samples.

### Sample quality control

First, we performed aCGH on a total of 550 DS samples. We performed three stages of sample/array quality control (QC) beginning with Agilent’s recommendations. Their recommended QC cutoff for arrays is a derivative log_2_ ratio (DLR) < 0.3. DLR is a measure of probe-to-probe noise and is the SD of adjacent probe log_2_ differences. Twenty-five samples failed to meet this threshold and were excluded (Supplemental Material, Figure S2 in File S2).

Second, while the remaining 525 microarrays met Agilent’s basic QC parameter of DLR < 0.3, visual inspection of log_2_ plots revealed a number of arrays with an increased probe variance. To quantitatively assess and account for this effect, we calculated the variances of intra-array probe log_2_ ratios to develop a conservative array inclusion criterion. We excluded 74 arrays with variance ≥ 1 SD over the mean from any further analysis (Figure S3 in File S2).

Third, to avoid biasing an individual microarray toward over- or undercalling gains or losses, it is important that the mean log_2_ ratio across the array is near the expected value of zero. The means of the intra-array probe log_2_ were calculated on the 451 remaining arrays (grand mean = −0.00045), and 25 arrays with individual means ≥ 2 SD from the group mean were removed (Figure S4 in File S2). After CNV detection (described below), we removed clear outlier samples that had the number of CNVs (deletions or duplications) called > 5 SD over the mean. This eliminated five samples.

To avoid spurious association results based on population stratification, we performed principal component analysis (PCA) on most of our samples that had genome-wide SNP data available from our previously published study (Ramachandran *et al.* 2015). Four samples without genotyping data were removed from further CNV analyses. In PLINK (version 1.9; Chang *et al.* 2015), SNPs were removed that had > 10% missingness or that failed the Hardy–Weinberg equilibrium exact test with a p-value < 1 × 10^−6^. Common SNPs with minor allele frequency (MAF) > 0.05 were pruned by PLINK’s “–indep-pairwise” command within 50 kb windows, a five SNP step, and an *r*^2^ threshold of 0.2, leaving 552,943 SNPs. The first five eigenvectors were calculated using the R package SNPRelate (Zheng *et al.* 2012) and plotted (Figures S5–S9 in File S2). PCA round 1 clearly separated self-identified African Americans from Caucasians. Round 2 was performed separately on African Americans and Caucasians. Six African Americans were visibly clear outliers (round 2 PC1 ≤ −0.127) and were removed from further analyses (Figures S6 and S8 in File S2). Five Caucasians were clear outliers (round 2 PC1 ≤ −0.1) and were also removed (Figures S7 and S9 in File S2). The final cohort contained 198 cases and 211 controls (Table S5 in File S3).

### CNV calling

We also evaluated the quality of data at the probe level. Because custom CGH arrays contain probes with unpredictable binding characteristics, the variances of normalized probe fluorescent signals were calculated, and 2193 probes with interarray variance ≥ 1 SD above the mean were removed (Figure S10 in File S2). These calculations were done on the full set of arrays passing the above DLR criteria and before the above intra- and interarray probe log_2_ variance calculations and filtering.

We used two algorithms, ADM2 and GADA, to identify putative CNVs (Pique-Regi *et al.* 2010). We required that CNVs be called by both algorithms to be included in the analysis. Parameters for Agilent’s ADM2 algorithm were set within their Genomic Workbench software (version 7.0.4.0) as follows: ≥ 6 probes, average log_2_ shift of ± 0.2, use of the diploid peak centralization, 2 kb window GC correction, intra-array replicates combined, and Fuzzy Zero applied. GADA adjustable parameters are the minimum probe number for a CNV to be called (MinSegLen) and a threshold, *T*_m_, referring to the minimum t-statistic that a predicted breakpoint must reach during its backward elimination procedure. We heuristically optimized the GADA *T*_m_ variable across a range of 4.5–20.5, by half steps, and evaluated performance based on two criteria: (1) whether the algorithm detected duplications ≥ 80% of our test samples at our known reference deletion, and (2) whether the algorithm detected common deletions found in the 1000 Genomes’ Phase 3 release of structural variants at a similar population frequency.

Compressed .vcf data the accompanying .tbi file for chromosome 21 produced from whole-genome sequencing by the 1000 Genomes Consortium was downloaded from http://hgdownload.cse.ucsc.edu/gbdb/hg19/1000Genomes/phase3/ on February 27, 2016. Tabix commands created unzipped .vcf files covering chr21:13,000,000–47,000,000, and variants denoted “SVTYPE” were filtered with shell commands, and variants denoted as deletion, duplication, or both (multi-allelic) were analyzed. The lower limit of CGH detection was set at ≥ 1798 bp, and variants of < 1798 bp were removed from the 1000 Genomes’ comparison set.

Of seven common CNVs on chromosome 21, our CGH array had at least six probes in two of these variants: esv3646598 and esv3646663. esv3646598 has a frequency of 0.064 in individuals of European ancestry (0.004 in African ancestry). esv3646663 has a frequency of 0.227 in individuals of African ancestry (0.001 in Europeans). We do not call absolute copy number from CGH data, and a deletion can represent zero, one, or two copies of the three expected. Thus, an upper frequency bound was set as Freq = (3(*d*) + 0(*N* − *d*))/3(*N*), while the lower bound was set as Freq = (1(*d*) + 0(*N* − *d*))/3(*N*), where *d* equals the number of times the deletion was called and *N* equals the total number of chromosomes. For both variants, in their respective ancestral population, *T*_m_ = 8 detects common structural variants within the expected range and also maximizes the detection of our reference deletion (Figures S11 and S12 in File S2). GADA was then launched using a custom R script applying the following parameters: estim.sigma2 = TRUE, MinSegLen = 6, and *T*_m_ = 0.8.

CNVs ≥ 1 Mb were removed (14 deletions; seven duplications) after visually checking log_2_ plots to confirm that these were likely false positives. Variants with breakpoints inside our reference deletion (chr21:45,555,257–45,615,042) were removed (0 deletions; 354 duplications). The p-arm and pericentromeric region of chromosome 21 are poorly mapped, and variants with breakpoints inside chr21:0–15,400,000 were removed (two deletions; one duplication). Clear outliers containing large numbers of deletions or duplications were removed. We used a threshold of > 5 SD over the mean of 0.73 deletions and 0.15 duplications calculated among the 426 arrays. Five SDs over the mean corresponded to > 5 deletions or two duplications within one array. These five samples contained 81 deletions and six duplications. The final data set includes 215 deletions and 59 duplications (Table S6 in File S3).

To assess the validity of our discovered CNVs, we compared them to CNVs discovered by other investigators in other cohorts registered in The Database of Genomic Variants (DGV: http://dgv.tcag.ca/). We used a custom script to test if each of our cohort’s CNVs had at least 50% reciprocal overlap with a DGV variant (http://dgv.tcag.ca/dgv/docs/GRCh37_hg19_variants_2015-07-23.txt).

### Replication study of Sailani *et al.* (2013)

Two common CNVs were found to be associated with DS+AVSD in the study by Sailani *et al.* (2013). To replicate these findings, we used the identical NanoString probes in 96 cases and controls from our DS cohort. We included probes that showed significant copy number differences between their cases and controls, totaling four of the eight probes for CNV1 and five of the seven probes for CNV2 (Table S7 in File S3). Samples were processed by the Gene Expression Analysis Laboratory at The University of Tennessee. We additionally used a different technology to replicate these findings. Two TaqMan (Applied Biosystems, Grand Island, NY) assays targeting each locus were selected for CNV1 and CNV2 (Table S7 in File S3). These assays were performed by the Emory Integrated Genomics Core on the same 96 samples tested by NanoString. Copy number calls were made by TaqMan’s CopyCaller software, and calls with a confidence probability < 0.8 were dropped.

### CNV association and burden analyses

We used PLINK v.1.07 to carry out association and burden analyses separately for deletions and duplications. To explicitly test the hypothesis that a gene reduced to two functional copies provides protection against AVSD in DS, we combined deletions that intersect an exon (refGene-hg19 updated July 3, 2016) with duplications that have predicted breakpoints within an exon to form a “reduced to disomy” set of CNVs. We also explicitly tested the inverse hypothesis: that genes entirely duplicated increase the risk for AVSD in DS. Three testing paradigms were performed: (1) burden analyses using the –cnv-indiv-perm and –cnv-count commands, (2) associations with individual CNV regions using –cnv-count, and (3) associations with individual genes overlapped by a CNV –cnv-intersect and –cnv-test-region. Empirical p-values of significance were determined by performing one million permutations for each test. These p-values are one-sided, and we tested for excess burden of duplications in cases and for deletions in controls. These three testing paradigms were applied to the full data set, as well as subsets of CNVs filtered by overlap in the DGV (downloaded January 2016), and by CNV frequency of greater than or less than 1% within out study population. Burden analysis in PLINK tests for differences between cases and controls using three different approaches: (1) is there a difference between the average number of CNVs per person (RATE); (2) is there a difference in the average number of bases covered by all CNVs (KBTOT); and (3) is there a difference in the average number of genes intersected by CNVs per person (GRATE)? We performed burden tests across deletions and duplications on chromosome 21 as entire sets and filtered by the allele frequency of the CNV (common or rare < 0.01) and by their existence or lack thereof in the DGV.

### Data availability

The Agilent design file for our custom array can be found in NCBI’s GEO database, accession number GPL22821. Raw array data are also in the GEO database, accession number GSE93004. Copy number variant calls are in NCBI’s dbVar database, accession study number nstd141.

## Results

We used rigorous quality control (see *Materials and Methods*) to identify deletions and duplications on the trisomic chromosome 21s in 409 DS individuals, including 355 Caucasians (174 DS+AVSD cases and 181 DS+NH controls) and 54 African Americans (24 DS+AVSD cases and DS+NH 30 controls). This analysis revealed a high-quality set of 215 individual deletions and 59 individual duplications (Table 1).

**Table 1:** CNV summary statistics for cases and controls stratified by race/ethnicity

		# Participants (Male/Female)	Type	# Variants	Average per Person	Size Range (kb)	Median Size (kb)
Caucasians	Cases DS+AVSD	77/97	Deletions	66	0.38	1.8–159.2	10.7
			Duplications	31	0.18	11.6–395.1	23.1
	Controls DS+NH	104/77	Deletions	71	0.39	1.8–239.1	10.7
			Duplications	23	0.13	10.4–200.0	18.0
African Americans	Cases DS+AVSD	6/18	Deletions	36	1.50	2.1–44.1	4.4
			Duplications	1	0.04	491.9	491.9
	Controls DS+NH	18/12	Deletions	42	1.40	2.1–260.3	7.2
			Duplications	4	0.13	4.5–14.4	10.9

Cases (DS+AVSD) are defined as those with Down syndrome and complete atrioventricular septal defect. Controls (DS+NH) are individuals with Down syndrome without a congenital heart defect.

### Validating CNV calls against the DGV

The DGV is a curated catalog of published peer-reviewed human structural variation (MacDonald *et al.* 2014, http://dgv.tcag.ca). Common CNVs discovered in our population that are also found in other populations registered in the DGV indicate a high likelihood of that CNV being a true positive. For Caucasians and African Americans, respectively, 125 of 137 (91%) and 78 of 78 (100%) of these deletions had 50% reciprocal overlap with deletions in the DGV. Of these, 44 of 54 (82%) Caucasian duplications and 3 of 5 (60%) in African American duplications were reported in the DGV. The size of discovered CNVs was not related to their presence in the DGV (Table S8 in File S3).

### No single CNV of large effect is associated with AVSD in DS

We performed association testing of single deletion and duplication regions along chromosome 21, as well as of single genes intersected by deletions or duplications, controlling for possible population stratification (see *Materials and Methods*). Though in Caucasians we had 80% power to detect risk variants of 5% allele frequency with an odds ratio of 2.2 or greater (α level of 0.05), no single CNV region was associated with AVSD (Figure S1 in File S2). We also tested for association of single genes with any intersection by CNVs and found no suggestive association. With our small African American cohort, we had 80% power to detect a risk CNV with an odds ratio of 6.3 at an allele frequency of 0.05 and an α level of 0.05. Again, we found neither a single CNV nor any CNV-intersected gene on chromosome 21 associated with AVSD in our DS population.

### Burden of chromosome 21 deletions

We tested our first hypothesis, that individuals with DS carrying chromosome 21 deletions may be protected from AVSD, because these deletions return AVSD susceptibility loci back to disomy. To do this, we compared the burden of chromosome 21 deletions among DS+AVSD cases to that of DS+NH controls. Using PLINK v1.07 (Purcell *et al.* 2007), we determined whether there was a greater average number of deletions per person measured in two ways: (1) as an increased number of bases covered by deletions and (2) as an increased average number of genes intersected by deletions on chromosome 21. We tested all deletions, filtered by allele frequency (common ≥ 0.01 or rare < 0.01) and whether or not they were reported in the DGV. Our analyses revealed no effect of deletions providing a protective effect against AVSDs in Caucasians (Table S1 in File S3). In contrast, African American DS+NH controls were significantly more likely to have more bases covered by deletions within the full deletion set (average total bases covered by deletions: 33.45 kb in DS+NH controls *vs.* 13.06 kb in DS+AVSD cases; empirical p-value = 0.04; Table S1 in File S3). When we filtered deletions by frequency, we found that this effect in African Americans was driven by rare variants of < 1% frequency in our study sample: African American DS+NH controls with rare deletions have, on average, 45.63 kb covered by rare deletions *vs.* 12.8 kb in DS+AVSD cases (empirical p-value = 0.02, Table S1 in File S3).

To further test our hypothesis, we redefined our definition of CNVs that might reduce a gene to disomy by disrupting gene function by including: (1) deletions that intersected an exon and (2) duplications that intersected an exon, but did not envelope an entire gene. In African Americans, this reduced the set to only two CNVs, a deletion and duplication in two controls (one-sided Fisher’s exact p-value = 0.32). In Caucasians, this produced a set of 41 CNVs in DS+AVSD cases and 41 CNVs in DS+NH controls that reduce a gene back to disomy; thus, there was no indication that individuals with DS without heart defects are protected by CNVs that reduce a gene back to disomy (Table S3 in File S3).

### Burden of chromosome 21 duplications

To test our second hypothesis, that chromosome 21 duplications increase the risk for AVSD, we compared the burden of chromosome 21 duplications among DS+AVSD cases compared with DS+NH controls. In Caucasians, a number of findings were consistent with this hypothesis (Table S2 in File S3). We observed that duplications, on average, affect more bases in cases (83.53 kb) than in controls (40.49 kb) (empirical p-value = 0.09). Caucasian cases also had twice the rate of genes duplicated compared with controls (0.22 in cases *vs.* 0.10 in controls; empirical p-value = 0.07). Rare CNVs in Caucasians drive these effects. For example, cases had a higher rate of rare duplications than controls (0.09 in cases *vs.* 0.03 in controls; empirical p-value = 0.06). More specifically, cases have five times the rate of genes intersected by rare duplications (0.16) compared with controls (0.03) (empirical p-value = 0.04). These effects remain by filtering for variants not in the DGV, as they are all rare variants. Given the low number of duplications in the African American samples, we did not see an increased burden of duplications among DS+AVSD cases (Table S2 in File S3).

Again, to further test this hypothesis, we filtered duplications for those that contained a full gene and found six in cases and one in a control (one-sided Fisher’s exact p-value = 0.10; odds ratio = 5.3, and 95% C.I. = 0.75–infinity). Two of these duplications reside in the same case individual.

### Replication of previous findings of common CNVs associated with DS-associated AVSD

We next sought to replicate two loci previously reported to be significantly associated with AVSD in a collection of individuals with DS (Sailani *et al.* 2013). CNV1 at chr21:43,193,374–43,198,244 (hg19) was observed as a deletion in 18% of the 55 cases with DS+AVSD and 0% of the 53 DS+NH controls, and as a duplication in 7% of cases and 0% in controls. CNV2 at chr21:43,411,411–43,413,231 was found as a deletion in 24% of controls *vs.* 0% in cases and as a duplication in 14% of cases *vs.* 11% of controls (Table 2). In an internal validation study based on 49 DS+AVSD cases and 45 DS+NH controls, Sailani *et al.* (2013) used NanoString nCounter technology and found significant differences in copy number ratios in probes targeting these loci.

**Table 2:** Comparison of DS+AVSD significantly associated CNVs from Sailani and co-workers to our current study

		Sailani and Co-workers’ CGH Results				Our CGH Results			
		Cases (*n* = 53)		Controls (*n* = 55)		Cases (*n* = 198)		Controls (*n* = 222)	
		DS+AVSD		DS+NH		DS+AVSD		DS+NH	
	Coordinates (hg19)	Deletion Frequency	Duplication Frequency	Deletion Frequency	Duplication Frequency	Deletion Frequency	Duplication Frequency	Deletion Frequency	Duplication Frequency
CNV1	chr21:43,193,374–43,198,244	0.18	0.07	0	0	0	0	0	0
CNV2	chr21:43,411,411–43,413,231	0.24	0.14	0	0.11	N/A	N/A	N/A	N/A

We did not replicate the previously reported significant association of common deletions and duplications at CNV1 in the study by Sailani *et al.* (2013). Our CGH array did not have at least six probes inside CNV2 and thus was undetectable by our methodology. CGH, comparative genomic hybridization; DS+AVSD, individuals with Down syndrome and complete atrioventricular septal defect; DS+NH, individuals with Down syndrome without a congenital heart defect (*i.e.*, normal heart); CNV, copy number variant; chr21, chromosome 21.

Our aCGH experiments had 19 probes within CNV1 and did not detect any CNV, though our sample size is four times larger than that of Sailani *et al.* (2013) (Table 2). Our custom array had only three probes in the CNV2 locus, and thus we were not able to detect it with our stringent criteria, which required six or more probes to call a CNV. We performed a NanoString experiment on a subset of our sample (49 cases and 45 controls) using the same CodeSet probes that Sailani *et al.* (2013) found to be significantly associated with DS+AVSD. We followed the same methodology they used to analyze the NanoString data. For each probe set, a ratio of the probe’s copy number count from a test individual over that of a reference DS sample was computed. A Mann–Whitney *U*-test was then applied at each probe, testing for a difference in the mean copy number count ratios between cases and controls. In CNV1, one of the three probes found significant in the Sailani *et al.* (2013) sample, we found a significant difference in nCounter copy number ratios between our cases and controls (p-value = 0.007) (Table 3). At CNV2, two of the six probes found to be significant in Sailani *et al.* (2013) were marginally significant in our data set (p-values = 0.054 and 0.056).

**Table 3:** Comparison of DS+AVSD significantly associated CNVs from Sailani and co-workers to our current study using NanoString technology

	Coordinates (hg19)	Sailani p-Value	Our Cohort p-Value
CNV1			
Probe 2	chr21:43,195,101–43,195,176	0.001	0.812
Probe 3	chr21:43,195,664–43,195,743	0.017	0.007
Probe 6	chr21:43,198,103–43,198,173	0.036	0.08
CNV2			
Probe 1	chr21:43,411,026–43,411,115	0.014	0.366
Probe 2	chr21:43,411,401–43,411,473	0.018	0.577
Probe 4	chr21:43,412,130–43,412,219	0.001	0.054
Probe 5	chr21:43,412,564–43,412,653	0.004	0.387
Probe 6	chr21:43,412,999–43,413,088	0.016	0.056
Probe 7	chr21:43,413,251–43,413,340	0.008	0.783

We performed NanoString nCounter assays on 46 DS+AVSD cases and 45 DS+NH controls using the same probes used by Sailani *et al.* (2013) in their CNV replication experiment that included 49 cases and 45 controls. To maintain congruency, we applied their assessment strategy to test for mean differences in normalized count (CN) ratios using a one-sided Mann–Whitney *U*-test. In CNV1, we detected a significant difference in CN ratios for probe 3 (using Sailani and co-workers’ nomenclature), but did not find this relationship for the other two probes previously found significant by Sailani *et al.* (2013). In CNV2, two of the six previously significant probes were marginally significant in our experiment. DS+AVSD, individuals with Down syndrome and complete atrioventricular septal defect; DS+NH, individuals with Down syndrome without a congenital heart defect (*i.e.*, normal heart); CNV, copy number variant; chr21, chromosome 21.

**Figure 1:**
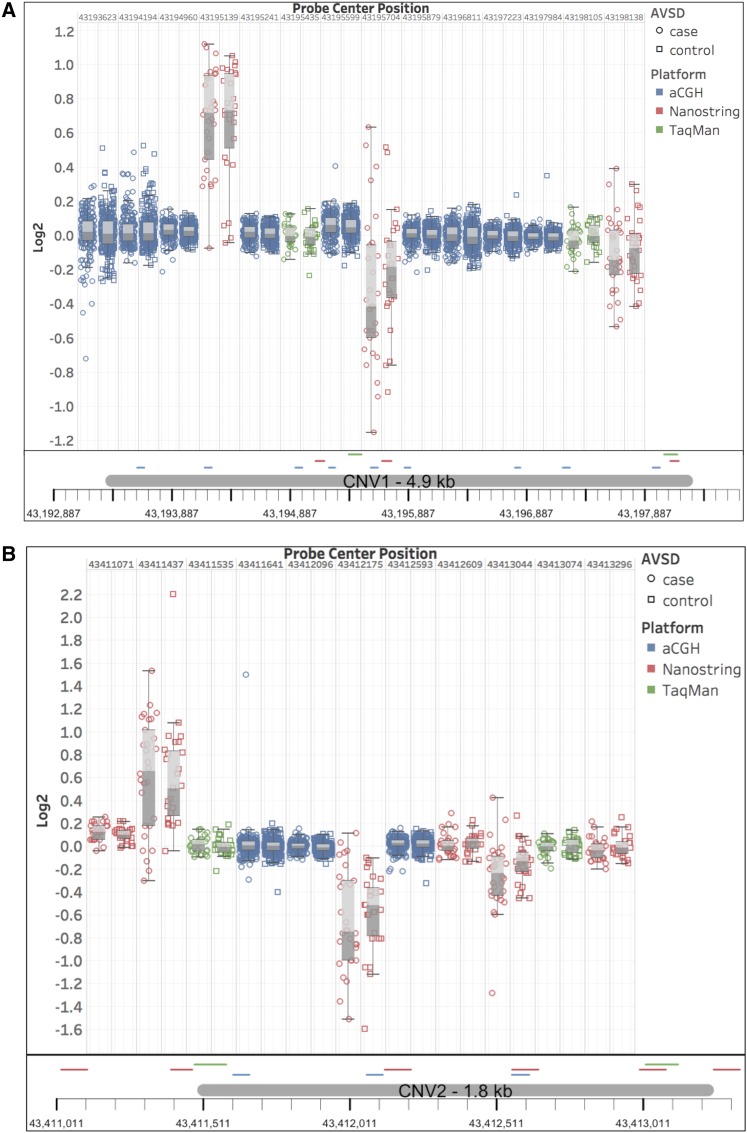
Summary results from analyses to attempt to replicate previously reported DS+AVSD (Down Syndrome with a complete atrioventricular septal defect)-associated common copy number variants (CNVs) with three technologies. (A and B) shows measures for Sailani-putative CNVs 1 and 2, respectively. Boxplots show inner quartile range of log_2_ ratios for tested DS samples with whiskers reaching 1.5 times the interquartile range. Case values are circles, and control values are in outlined boxes. Array comparative genomic hybridization (aCGH) probes are blue, NanoString probes are red, and TaqMan probes are green. Boxplots are ordered by genomic location, and their precise locations are indicated beneath the plots. Neither CGH probes nor TaqMan Copy Number assays detected aberrant copy numbers or differences between cases and controls. Varying results were found across these loci by NanoString probes, with some probes showing differences in log_2_ means between cases and controls. Within these same proposed small CNV loci, NanoString probes called all possible combinations of copy gain, loss, and no change within the same small cohort. When compared to adjacent CGH and TaqMan probes, it is clear that the NanoString probes are not reliable predictors of copy number state at this locus.

The mixed results within NanoString and aCGH experiments led us to assess the validity of these findings with a third technology (Figure 1). Two TaqMan probe sets were selected within each CNV and tested in 46 DS+AVSD cases and 46 DS+NH controls, including the same cases and controls analyzed with NanoString. No deletions were identified by either probe set in CNV1 or CNV2 (Table S4 in File S3). At CNV1, a duplication was detected in one individual (DS+NH control) by one probe set; the other did not detect a copy number change. At CNV2, a duplication was detected in one individual (a DS+NH control) by both probe sets. No DS+AVSD cases had copy number calls at either CNV.

## Discussion

Our cohort of individuals with DS with complete AVSD and those with structurally normal hearts represents the largest study of its kind to date. In addition, our CNV data set was built applying conservative quality control metrics on probe, array, and sample inclusion, yielding robust conclusions after analysis. The composite set of CNVs, which required concordance between two well-established CNV calling algorithms, generated a data set with a low likelihood of false-positive findings, as indicated by their high representation in the DGV (94% of deletions and 80% of duplications).

Given our large sample size for this relatively rare condition, we had 80% power to detect a single CNV at 5% population frequency with an odds ratio of ≥ 2.2 in Caucasians and 6.3 in African Americans (empirical p-value of 0.05). We detected no single CNV with an effect size of this magnitude. Our data suggest that it is unlikely for a single common variant > 1.8 kb on chromosome 21 to explain the 2000-fold increased risk for AVSD on a trisomy 21 background. This is consistent with our previous findings (Ramachandran *et al.* 2015a,b). We also performed gene ontology and gene-set enrichment analyses (read File S1). Both analyses hint at possible perturbation of ciliary pathways, but the small numbers limit further interpretation.

We were unable to replicate two previously reported common CNVs on chromosome 21 associated with DS+AVSD in a smaller cohort by Sailani *et al.* (2013). Although our custom array did not have enough probes to reliably detect CNV2, we were powered to detect CNV1 and did not find this CNV in either cases or controls in our larger population. Sailani *et al.* (2013) replicated their finding in an independent sample by reporting differences in means of NanoString nCounter probe ratios between cases and controls. At CNV1, we tested three of these significant probe sets with available coordinates and found only one to be significant by Mann–Whitney *U*-test. At CNV2, we tested the six probes that were previously found to be significant and found two of the six to be marginally significant. Sailani *et al.* (2013) calculated ratios of probe counts of the test sample over that of the reference sample, and tested for ratio differences between cases and controls using a one-sided Mann–Whitney *U*-test. We applied their techniques to a similar sized subset of our larger cohort and found inconclusive results to support their findings. As a final validation of these proposed DS+AVSD-associated CNVs, we performed TaqMan Copy Number assays with two probe sets for each CNV. As detailed in the *Results*, no deletions were detected at CNV1 or CNV2. Thus, we failed to replicate their reported findings for CNV1 in a cohort that was four times larger, and for both CNV1 and CNV2 using a similar technology and a follow-up gold-standard technology (Figure 1).

Our study stands in agreement with the consensus of other studies reporting complex heterogeneity of genetic contributions to atrioventricular septum and valve development in both the disomic population and in individuals with trisomy 21 (Robinson *et al.* 2003; Ackerman *et al.* 2012; Al Turki *et al.* 2014; Ramachandran *et al.* 2015a,b; Priest *et al.* 2012). Our data support the nuanced hypotheses that deletions on chromosome 21 on a trisomic background reduce the risk for AVSD and duplications on chromosome 21 further increase risk of AVSD in DS. These effects were enriched when considering rare (MAF < 0.01) deletions and duplications. Rare deletions have been previously implicated within our DS cohort, where DS+AVSD cases were found to have a greater genome-wide burden of rare, large (> 100 kb) deletions (Ramachandran *et al.* 2015a,b)

Moving forward, genetic studies of CHD in DS, as well as nonsyndromic CHDs, should be designed with this considerable genetic heterogeneity in mind. It is clear that, while trisomy 21 alone increases the risk for AVSD 2000-fold, its probable mode of action is through epistatic interactions among many genes. Untangling these complex risk factors will require a larger cohort of individuals with DS with and without CHDs to find susceptibility loci of measurable effect. As these cohorts continue to grow, efforts should focus on exome and whole-genome sequencing approaches that identify rare variants, whose effects can be tested for burdening candidate genetic pathways of cardiogenesis. Finally, environmental factors require greater consideration, and resources should be prioritized to gather broad epidemiological data and link them to genomic resources.

## Supplementary Material

Supplemental material is available online at www.g3journal.org/lookup/suppl/doi:10.1534/g3.117.300366/-/DC1.
