# Supplementary material for: Analysis of Copy Number Variants on Chromosome 21 in Down Syndrome-Associated Congenital Heart Defects
Source: G3 (Bethesda). 2017 Nov 15;8(1):105–11. doi: 10.1534/g3.117.300366 (PMC5765339; doi:10.1534/g3.117.300366)
Supplement: Supplementary file 1 [file 105FileS1.docx]

**Gene Set Enrichment and Gene Ontology Term Analyses**

**Methods**

We used PLINK v1.07 to perform Gene Set Enrichment Analysis (GSEA) (Raychaudhuri et al. 2010) on a set of cilia-related genes (Table 1) compiled by McClintock et al. 2008 and previously implicated in Down syndrome-associated AVSD (Ripoll et al. 2012; Ramachandran et al. 2014; Li et al. 2015). GSEA tests the hypothesis that cilia genes are enriched for CNVs compared to all chromosome 21 genic CNVs and assigns a one-sided empirical p-value by one million permutations, indicating positive enrichment in cases for duplications or in controls for deletions.

We performed a Gene Ontology (GO) term analysis using the ClueGO Extension in Cytoscape v.3.3.0 (Bindea et al. 2009). Gene lists were created based on those intersected by deletions or duplications only in cases or only in controls (Table 2). Each gene set was analyzed with the following settings: Ontologies = Go-Biological Process downloaded May 5^th^, 2016, Evidence = All, Pathway Significance = 0.05, GO Tree Interval = 3 minimum level and 13 maximum level, two gene minimum or 1% of pathway genes, and Bonferroni step-down p-value correction with mid-P-values. Other parameters were left at default settings.

| **Gene** | **Coordinates (hg19)** |
| --- | --- |
| GART | chr21:34876237-34915198 |
| MCM3AP | chr21:47655047-47705236 |
| RSPH1 | chr21:43892596-43916401 |
| ABCG1 | chr21:43619798-43717354 |
| DYRK1A | chr21:38739858-38887679 |
| PWP2 | chr21:45527207-45551063 |
| PCNT | chr21:47744035-47865682 |
| SOD1 | chr21:33031934-33041243 |
| HSPA13 | chr21:15743436-15755509 |
| USP25 | chr21:17102495-17252377 |
| U2AF1 | chr21:44513065-44527688 |
| WDR4 | chr21:44263203-44299678 |
| CCT8 | chr21:30428647-30446010 |
| CBS | chr21:44473300-44496040 |
| PDXK | chr21:45138977-45182188 |
| COL18A1 | chr21:46825096-46933634 |
| ITSN1 | chr21:35014783-35261609 |

Table 1: Cilia gene list used for Gene Set Enrichment Analysis. Assembled from Ripoll et al. 2012.

| **African Americans** | | | | **Caucasians** | | | |
| --- | --- | --- | --- | --- | --- | --- | --- |
| **Deletions** | | **Duplications** | | **Deletions** | | **Duplications** | |
| **Case only** | **Control only** | **Case only** | **Control only** | **Case only** | **Control only** | **Case only** | **Control only** |
| LOC339622 | C21orf56 | ATP5J | SIK1 | AGPAT3 | CHODL | ADAMTS5 | CBR3 |
|  | COL6A1 | GABPA |  | MIR802 | DYRK1A | C21orf49 | CBR3-AS1 |
|  | COL6A2 | JAM2 |  | PDE9A | LINC00308 | C21orf62 | DOPEY2 |
|  | FTCD | LINC00158 |  | SAMSN1 | LINC00478 | CRYZL1 | LOC100133286 |
|  | HLCS | LINC00515 |  | TRPM2 | LINC00515 | DONSON | SCAF4 |
|  | LSS | MIR155 |  |  | PCP4 | DSCAM |  |
|  | PCBP3 | MIR155HG |  |  | PDXK | DSCAM-AS1 |  |
|  |  | MRPL39 |  |  | PKNOX1 | GCFC1 |  |
|  |  |  |  |  | TCP10L | GCFC1-AS1 |  |
|  |  |  |  |  |  | ITSN1 |  |
|  |  |  |  |  |  | KRTAP21-1 |  |
|  |  |  |  |  |  | KRTAP21-2 |  |
|  |  |  |  |  |  | KRTAP21-3 |  |
|  |  |  |  |  |  | KRTAP7-1 |  |
|  |  |  |  |  |  | KRTAP8-1 |  |
|  |  |  |  |  |  | NCAM2 |  |
|  |  |  |  |  |  | PRMT2 |  |
|  |  |  |  |  |  | S100B |  |
|  |  |  |  |  |  | SON |  |
|  |  |  |  |  |  | SYNJ1 |  |
|  |  |  |  |  |  | USP25 |  |
|  |  |  |  |  |  | ZNF295 |  |

Table 2. Genes provided to ClueGO for GO term pathway analysis. In each list, genes intersected by the respective CNV type only in DS+AVSD cases or only in DS+NH controls were submitted for pathway analysis. Respective CNV type + case/control status gene lists were combined between the two populations.

**Results**

Previous reports suggest that genetic variation in cilia genes plays a role in AVSD in DS (Ripoll et al. 2012; Ramachandran et al. 2015; Burnicka-Turek et al. 2016). There are 19 genes on chromosome 21 implicated as part of the ciliome (Supplemental Table 9, McClintock et al., 2008). We performed Gene Set Enrichment Analysis (GSEA) with PLINK v1.07 (Purcell et al. 2007) to test two hypotheses: 1) chromosome 21 deletions are more likely to intersect cilia genes than other genes in DS+NH controls and 2) chromosome 21 duplications are more likely to intersect cilia genes than other genes in DS+AVSD cases. In Caucasians, two putative cilia genes, *DYRK1A* and *PDXK,* were intersected by deletions in controls; no cilia genes were intersected by deletions in cases (Table 3). While the counts are low, this finding is suggestive (permuted p-value = 0.1) by GSEA. Similarly, for duplications, we observed the inverse: duplications intersect with two cilia genes, *USP25* and *ITSN1*, in cases and none in controls (permuted p-value = 0.2). When we combined deletions and duplications that disrupt exons and thus reduce that gene to disomy, we found one case and two controls with a CNV disrupting a cilia gene exon (permuted p-value = 0.25).

|  | **Number of cilia genes intersected** | | |
| --- | --- | --- | --- |
|  | **Cases DS+AVSD** | **Controls DS+NH** | **p-value** |
| **Deletions** | 0 | 2 | 0.1 |
| **Duplications** | 2 | 0 | 0.2 |

Table 3. Counts of cilia genes on chromosome 21 intersected by CNVs. Gene Set Enrichment Analysis (GSEA) tests for enrichment of CNVs in 19 cilia genes compared to all other genic CNVs. Two cilia genes are intersected by deletions in controls, which suggests an enrichment from a GSEA permuted p-value of 0.1. We find the opposite pattern in duplications where two genes are intersected only in controls (GSEA permuted p-value of 0.2). Although not statistically significant, the pattern is consistent with our stated hypothesis.

To uncover novel pathways disrupted by CNVs in DS-associated AVSD, we performed a Gene Ontology analysis with the ClueGO v2.2.5 (Bindea et al. 2009) plugin in Cytoscape v3.3.0 (Shannon et al. 2003), providing lists of genes that were intersected by deletions and duplications only in cases or only in controls (Supplemental Table 4). As we assume the same pathways leading to AVSD will be perturbed in all humans, we combined lists of genes from African Americans and Caucasians, creating eight gene lists: 1. genes intersected by deletions only in cases or 2. only in controls; 3. genes intersected by duplications only in cases or 4. only in controls; 5. genes with an exon intersected by a deletion or non-gene-enveloping duplication only in cases or 6. only in controls; and 7. genes completely duplicated only in cases or 8. only in controls. In the deletion gene lists, only those within DS+NH controls clustered into a pathway. Deleted genes in controls were overrepresented in protein heterotrimerization (GO:0070208, p-value = 0.0002). In the duplication gene lists, significant pathway enrichment was found only in DS+AVSD cases. These duplication-intersected genes were significantly enriched for synaptic vesicle endocytosis (GO:0048488, p-value = 0.0001). While genes with exons disrupted by CNVs in cases showed no enrichment in biological pathways, those in controls were enriched in protein heterotrimerization (GO:0070208, p-value = 0.0047). There was only one gene and one ncRNA completely duplicated in controls, while there were 19 genes duplicated in cases, and they were enriched in the process of histone methylation (GO:0016571, p-value = 0.0017).

**Discussion**

We specifically examined genes required for proper cilia function because of the mounting evidence for their involvement in CHD. We found a suggestive association of perturbations in such genes, consistent with our previous genome-wide results in this same cohort, where we found an increased burden of deletions overlapping cilia genes in cases versus controls (Ramachandran et al. 2015). Other support for ciliome involvement comes from multiple lines of evidence. In a forward genetic mouse screen, 87,355 fetuses from mutagenized mice resulted in 218 mice with CHDs (Li et al. 2015). Exome sequencing of 113 of these mice revealed 91 recessive mutations in 61 genes, of which 34 were involved in cilia function. Disruption of cilia-related genes is also linked to AVSD in lymphoblastoid cell lines (LCLs) from individuals with DS (Ripoll et al. 2012). Comparing gene expression profiles of LCLs from individuals with DS without CHDs to those with atrial septal defects (ASDs), ventricular septal defects (VSDs), or AVSDs, they found significant deregulation of cilia genes within the AVSD group. Furthermore, principal component analysis of the expression profiles separate individuals with AVSDs from those with ASDs or VSDs, pointing to a different etiology of disease progression and substantiating the need to study phenotypically distinct CHDs independently. Most recently, Burnicka-Turek et al. characterized independent mouse lines with deleterious non-synonymous mutations induced in the cilia genes *Dnah11* and *Mks1* that caused AVSD (Burnicka-Turek et al. 2016).

**References**

BINDEA G., MLECNIK B., HACKL H., CHAROENTONG P., TOSOLINI M., KIRILOVSKY A., FRIDMAN W.-H., PAG√®S F., TRAJANOSKI Z. & GALON J. 2009. ClueGO: a Cytoscape plug-in to decipher functionally grouped gene ontology and pathway annotation networks. *Bioinformatics* 25: 1091-1093.

BURNICKA-TUREK O., STEIMLE J.D., HUANG W., FELKER L., KAMP A., KWEON J., PETERSON M., REEVES R.H., MASLEN C.L., GRUBER P.J., YANG X.H., SHENDURE J. & MOSKOWITZ I.P. 2016. Cilia gene mutations cause atrioventricular septal defects by multiple mechanisms. *Human Molecular Genetics*

LI Y., KLENA N.T., GABRIEL G.C., LIU X., KIM A.J., LEMKE K., CHEN Y., CHATTERJEE B., DEVINE W., DAMERLA R.R., CHANG C., YAGI H., SAN AGUSTIN J.T., THAHIR M., ANDERTON S., LAWHEAD C., VESCOVI A., PRATT H., MORGAN J., HAYNES L., SMITH C.L., EPPIG J.T., REINHOLDT L., FRANCIS R., LEATHERBURY L., GANAPATHIRAJU M.K., TOBITA K., PAZOUR G.J. & LO C.W. 2015. Global genetic analysis in mice unveils central role for cilia in congenital heart disease. *Nature*

McClintock, T. S., Glasser, C. E., Bose, S. C., & Bergman, D. a. 2008. Tissue expression patterns identify mouse cilia genes. *Physiological Genomics*, *32*(2), 198–206. http://doi.org/10.1152/physiolgenomics.00128.2007

RAMACHANDRAN D., MULLE J.G., LOCKE A.E., BEAN L.J.H., ROSSER T.C., BOSE P., DOOLEY K.J., CUA C.L., CAPONE G.T., REEVES R.H., MASLEN C.L., CUTLER D.J., SHERMAN S.L. & ZWICK M.E. 2014. Contribution of copy-number variation to Down syndrome-associated atrioventricular septal defects. *Genetics in medicine : official journal of the American College of Medical Genetics* 1-7.

RAYCHAUDHURI S., KORN J.M., MCCARROLL S.A., ALTSHULER D., SKLAR P., PURCELL S. & DALY M.J. 2010. Accurately assessing the risk of schizophrenia conferred by rare copy-number variation affecting genes with brain function. *PLoS genetics* 6:

RIPOLL C., RIVALS I., AIT YAHYA-GRAISON E., DAUPHINOT L., PALY E., MIRCHER C., RAVEL A., GRATTAU Y., BL’EHAUT H., M\’EGARBANE A., DEMBOUR G., DE FR\’EMINVILLE B., TOURAINE R., CR’EAU N., POTIER M.C. & DELABAR J.M. 2012. Molecular signatures of cardiac defects in down syndrome lymphoblastoid cell lines suggest altered ciliome and hedgehog pathways. *PloS one* 7: e41616.

SHANNON P., MARKIEL A., OZIER O., BALIGA N.S., WANG J.T., RAMAGE D., AMIN N., SCHWIKOWSKI B. & IDEKER T. 2003. Cytoscape: A Software Environment for Integrated Models of Biomolecular Interaction Networks. *Genome Research* 13: 2498-2504.
