# Supplementary material for: Analysis of Copy Number Variants on Chromosome 21 in Down Syndrome-Associated Congenital Heart Defects
Source: G3 (Bethesda). 2017 Nov 15;8(1):105–11. doi: 10.1534/g3.117.300366 (PMC5765339; doi:10.1534/g3.117.300366)
Supplement: Supplementary file 2 [file 105FileS2.docx]

**Supplemental Figures**


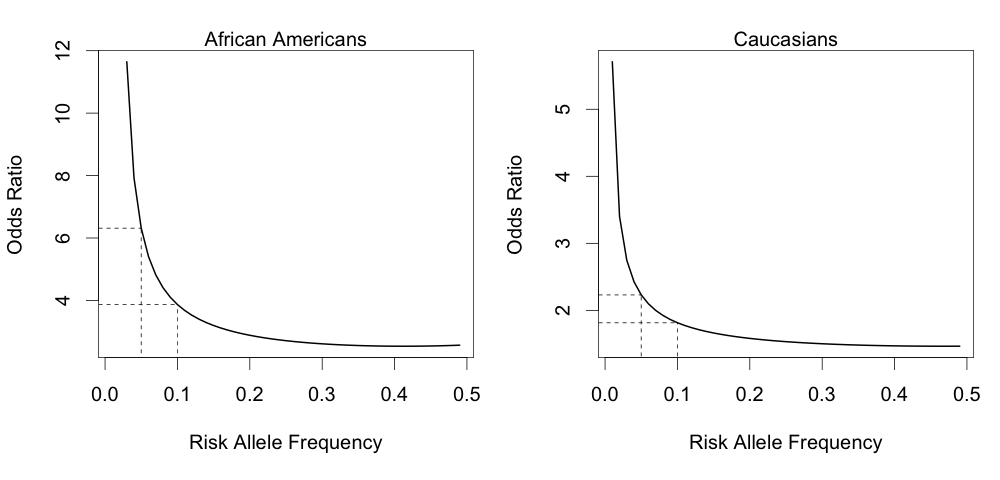


Figure S1. Power calculations assuming a nominal p-value = 0.05, additive model, and disease frequency of 0.18. We had 80% power to detect a risk variant with allele frequency = 0.05 with odds ratio (OR) of 6.3 and with an OR of 3.9 at allele frequency = 0.1. In Caucasians, we had 80% power to detect ORs of 2.2 and 1.8 for risk allele frequencies of 0.05 and 0.1, respectively.


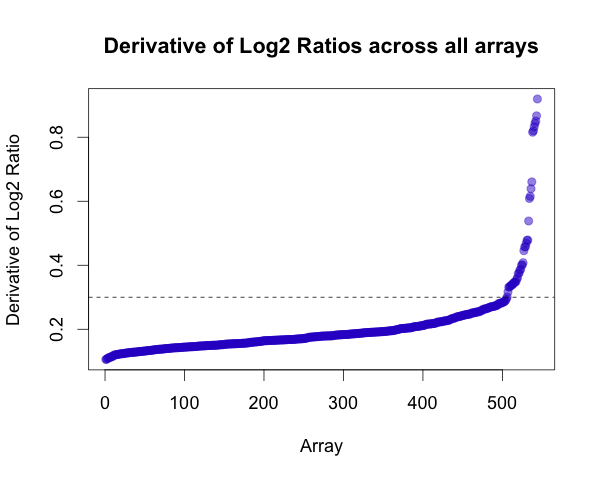


Figure S2. Derivative of log2 ratios (DLR) were calculated within Agilent’s Feature Extraction software, and arrays with DLR ≥0.3 were removed from further analyses.


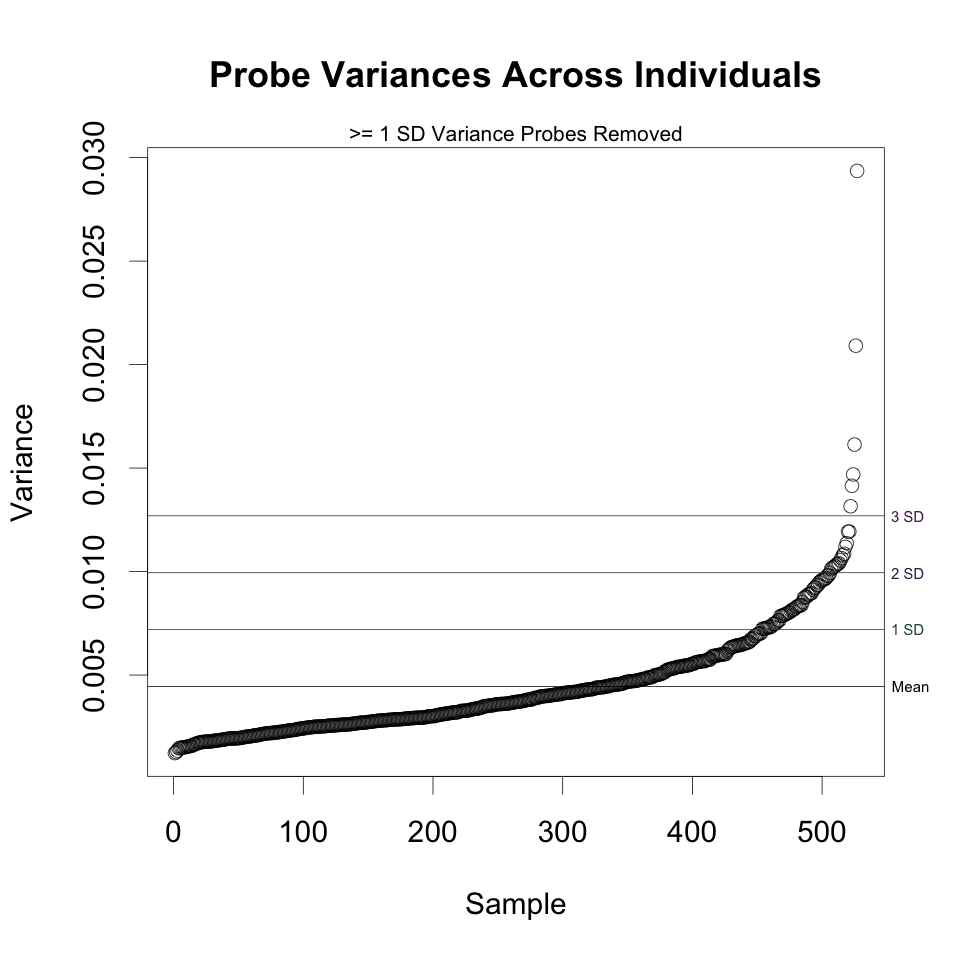


Figure S3. 74 arrays with intra-array probe log2 variance ≥one standard deviation over the mean were removed from further analyses.


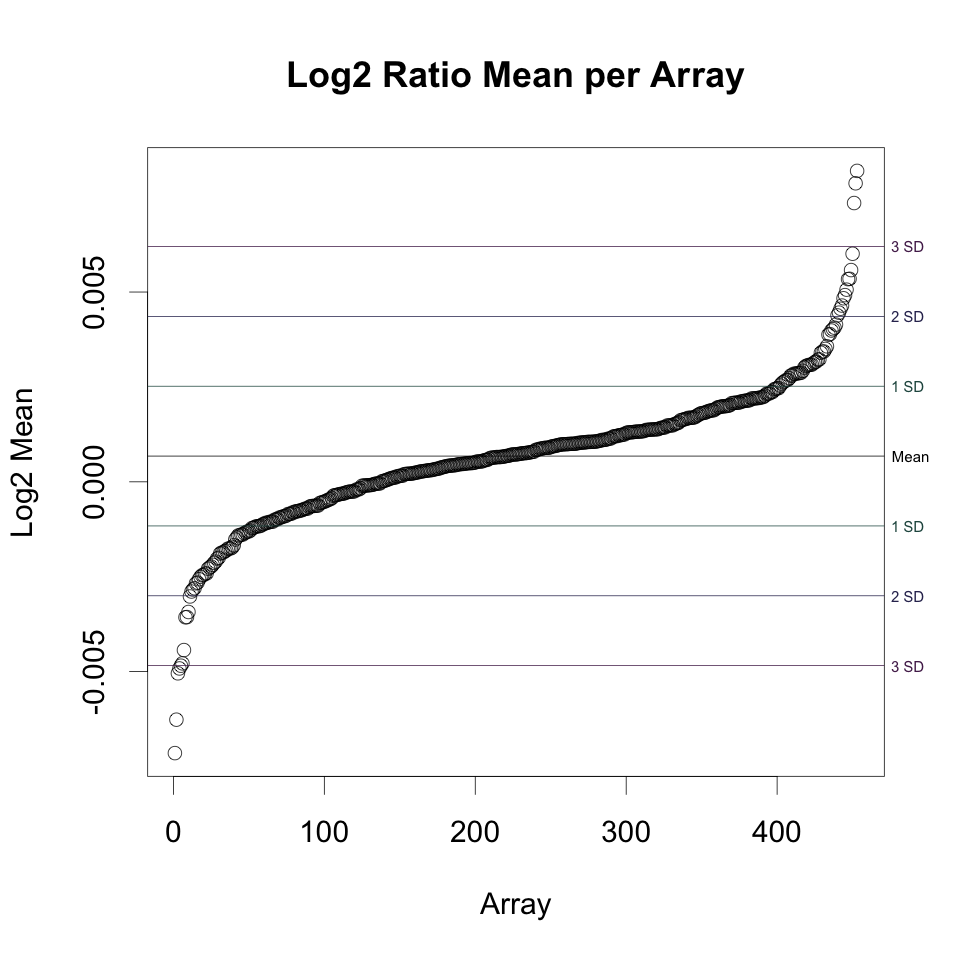


Figure S4. 25 arrays with intra-array log2 probe means ≥2 standard deviations from the grand mean were removed from further analyses.


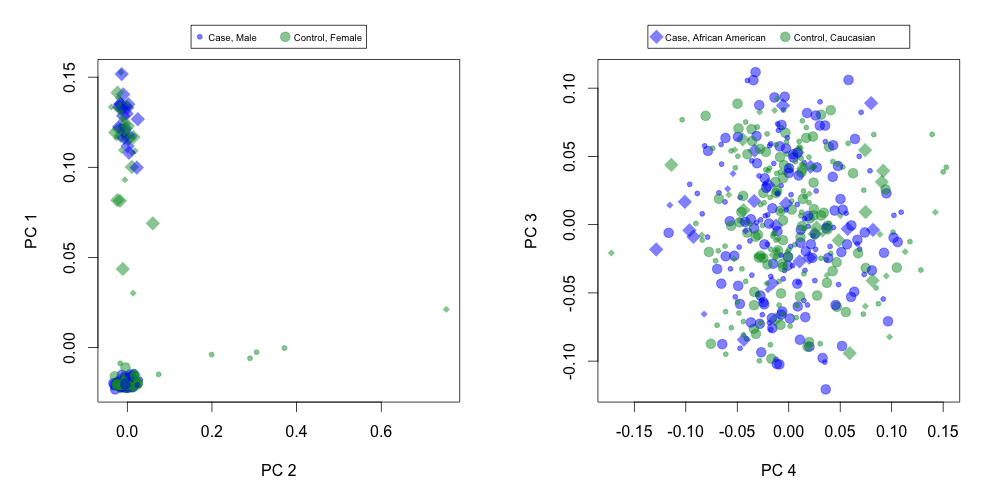


Figure S5. Round 1 of principal component analysis properly separates self-identifying African Americans from Caucasians.


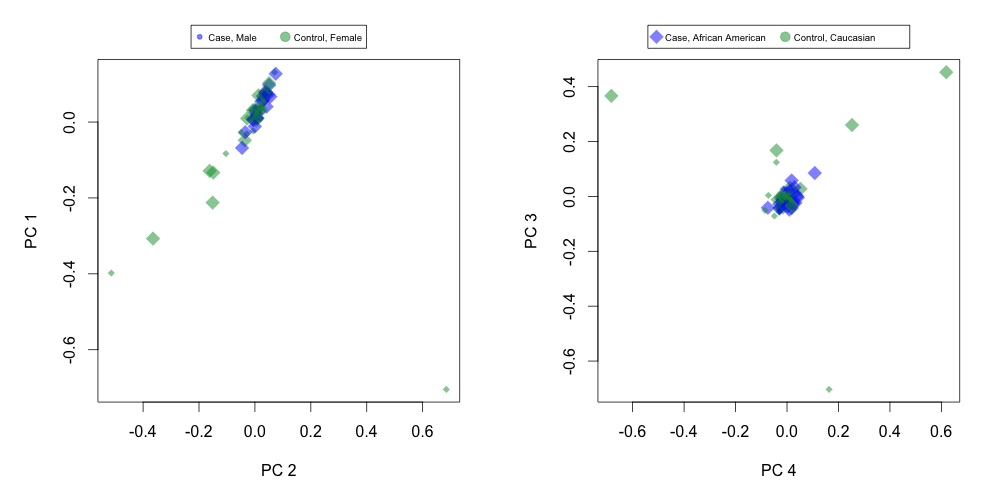


Figure S6. Round 2 of principal component analysis in African Americans shows population outliers. African American individuals with PC1 eigenvalues <-0.127 were removed from further analyses.


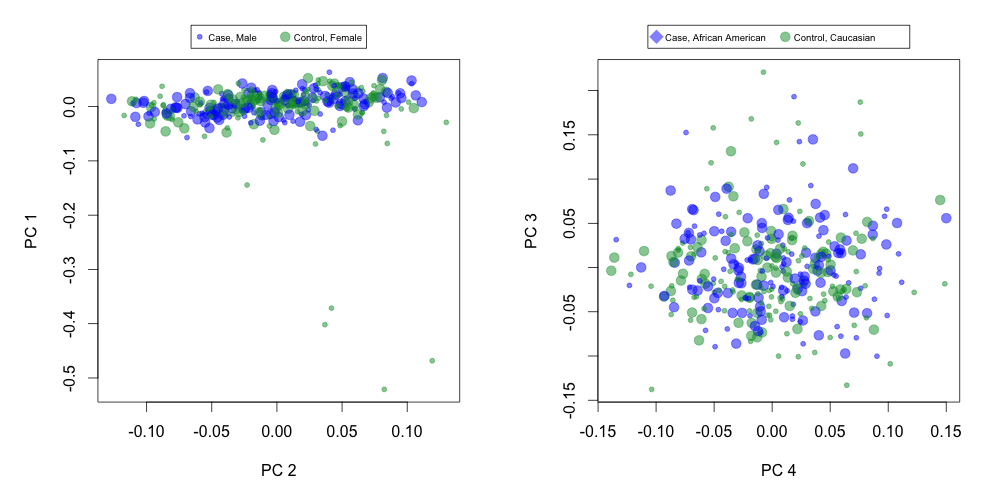


Figure S7. Round 2 of principal component analysis in Caucasians shows population outliers. Caucasian individuals with PC1 eigenvalues <-0.1 were removed from further analyses.


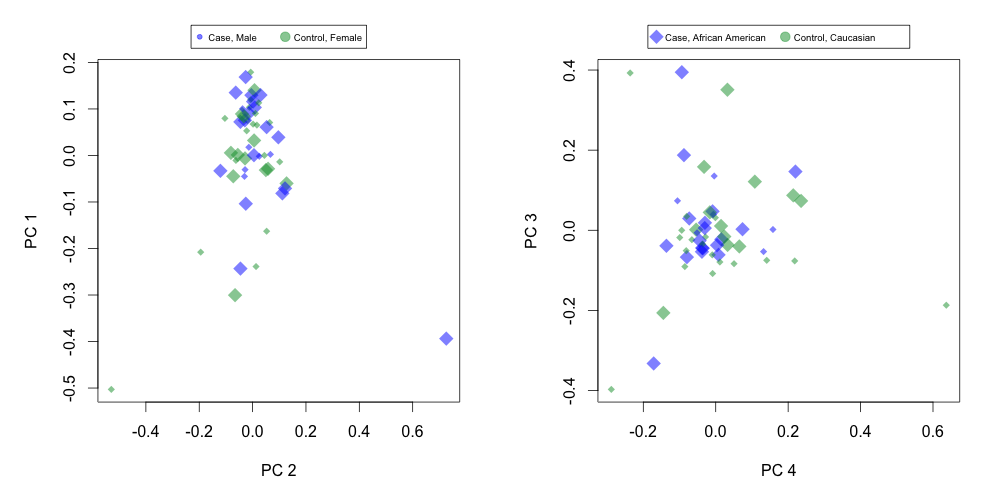


Figure S8. Final principal component analysis plots of African Americans show random scattering of individuals without clear case/control biases.


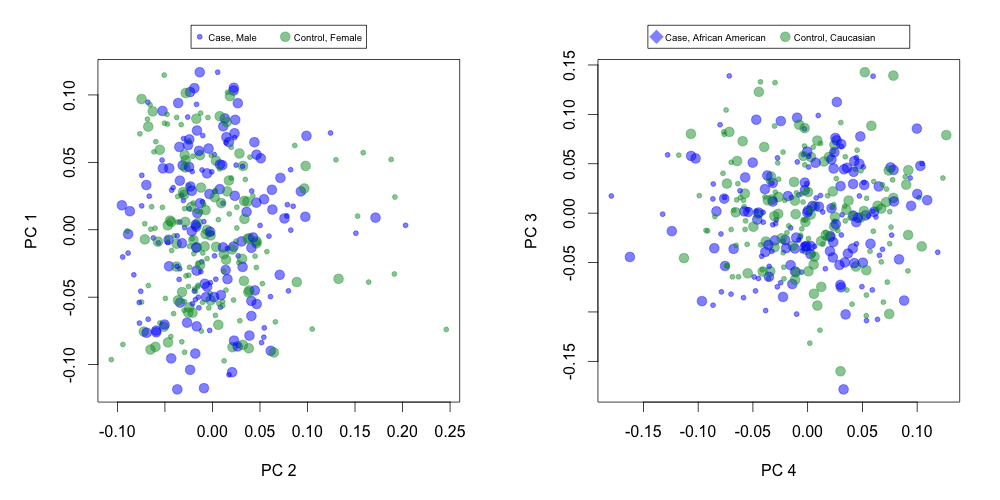


Figure S9. Final principal component analysis plots of Caucasians show random scattering of individuals without clear case/control biases.


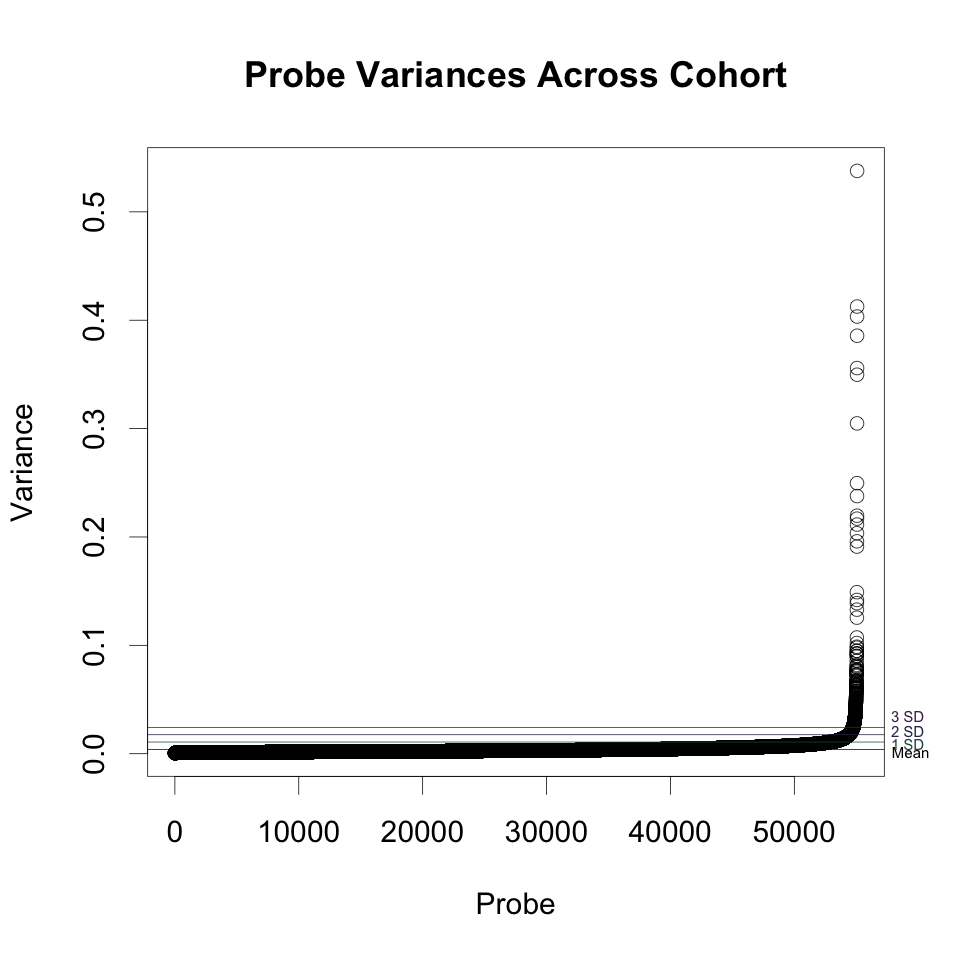


Figure S10. Data from 2,193 probes with an inter-array normalized fluorescence log2 ratio variance ≥ 1 standard deviation over the mean were removed.


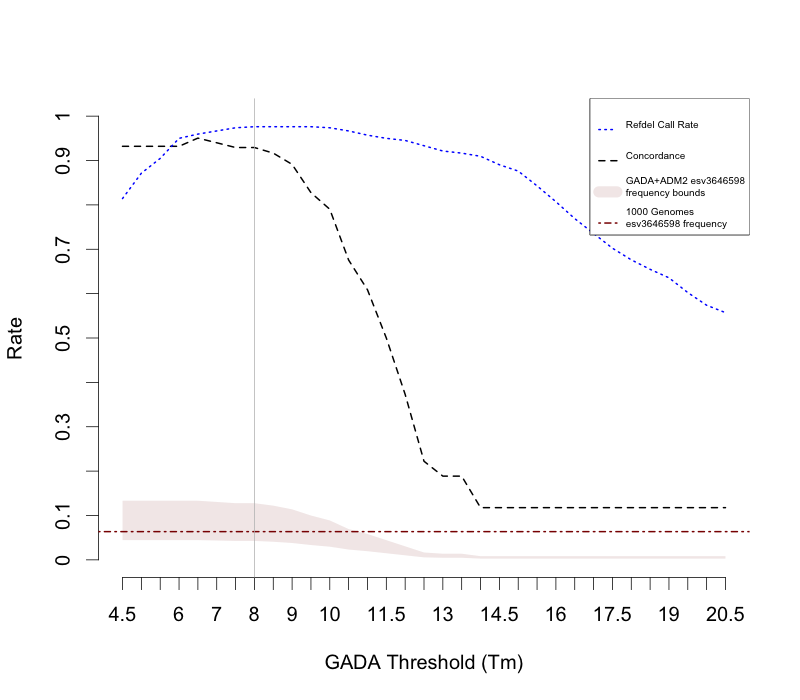


Figure S11. 1000 Genomes deletion esv3646598 detection across GADA thresholds. To optimize the GADA threshold parameter (T_m_) for calling CNVs, we aimed to optimize the detection of a known deletion (chr21:45,555,257-45,615,042) in our reference sample (called as a duplication in test samples) and to call the common deletion esv3646598 that was reported in 1000 Genomes Phase 3 Structural Variant release at a frequency of 0.0636 (red dash-dot line) in individuals of European ancestry. The dotted blue line represents the consensus call rate of our reference deletion by ADM2 + GADA. The dashed black line is the concordance rate of ADM2 + GADA calling esv3646598. The red highlighted area displays the estimated frequency bounds of the esv3646598 deletion as detected by the two algorithms across increasing GADA T_m_. We chose a GADA T_m_ of 8, which maximized our reference deletion call rate and called esv3646598 within the expected range.


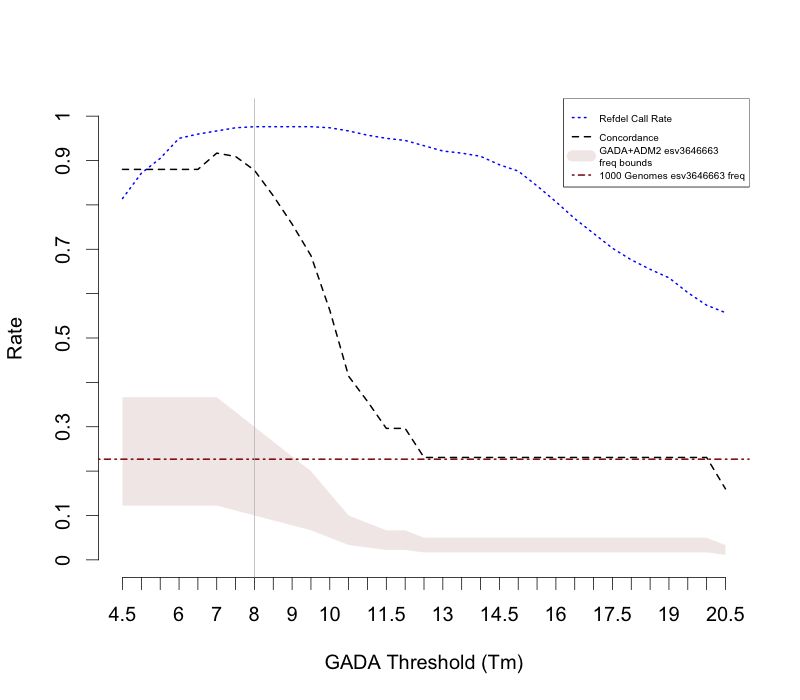


Figure S12. 1000 Genomes deletion esv3646663 detection across GADA thresholds. To optimize the GADA threshold parameter (T_m_) for calling CNVs, we aimed to optimize the detection of a known deletion (chr21:45,555,257-45,615,042) in our reference sample (called as a duplication in test samples) and to call the common deletion esv3646663 that was reported in 1000 Genomes Phase 3 Structural Variant release at a frequency of 0.2269 (red dash-dot line) in individuals of European ancestry. The dotted blue line represents the consensus call rate of our reference deletion by ADM2 + GADA. The dashed black line is the concordance rate of ADM2 + GADA calling esv3646663. The red highlighted area displays the estimated frequency bounds of the esv3646663 deletion as detected by the two algorithms across increasing GADA T_m_. We chose a GADA T_m_ of 8, which maximized our reference deletion call rate and called esv3646663 within the expected range.
