# Supplementary material for: Analysis of Copy Number Variants on Chromosome 21 in Down Syndrome-Associated Congenital Heart Defects
Source: G3 (Bethesda). 2017 Nov 15;8(1):105–11. doi: 10.1534/g3.117.300366 (PMC5765339; doi:10.1534/g3.117.300366)
Supplement: Supplementary file 3 [file 105FileS3.docx]

**Supplemental Tables**

|  |  | **Caucasian** | | | **African American** | | |  |
| --- | --- | --- | --- | --- | --- | --- | --- | --- |
| **Filtering** | **Burden test** | **DS+AVSD**  **cases (n = 174)** | **DS+NH**  **controls (n = 181)** | **Empirical permuted p-value** | **DS+AVSD**  **cases (n = 24)** | **DS+NH**  **controls (n = 30)** | **Empirical permuted p-value** | **Combined p-value** |
| **All** | **Number of deletions** | 66 | 71 | N/A | 36 | 42 | N/A |  |
|  | **Average number of deletions** | 0.38 | 0.39 | 0.46 | 1.50 | 1.40 | 0.67 | 0.67 |
|  | **Average kb covered by deletions** | 31.24 | 37.95 | 0.23 | 13.06 | 33.45 | **0.04** | **0.05** |
|  | **Avg. number of chr21 genes intersected by duplications** | 0.13 | 0.14 | 0.44 | 0.13 | 0.30 | 0.42 | 0.49 |
| **Common**  **(≥0.01)** | **Number of deletions** | 45 | 46 | N/A | 19 | 18 | N/A |  |
|  | **Average number of deletions** | 0.26 | 0.25 | 0.58 | 0.79 | 0.60 | 0.88 | 0.86 |
|  | **Average kb covered by deletions** | 29.52 | 28.04 | 0.60 | 5.57 | 6.94 | 0.09 | 0.22 |
|  | **Avg. number of chr21 genes intersected by duplications** | 0.09 | 0.07 | 0.76 | 0.00 | 0.00 | 1.00 | 0.97 |
| **Rare**  **(<0.01)** | **Number of deletions** | 21 | 25 | N/A | 17 | 24 | N/A |  |
|  | **Average number of deletions** | 0.12 | 0.14 | 0.39 | 0.71 | 0.80 | 0.42 | 0.46 |
|  | **Average kb covered by deletions** | 26.01 | 42.41 | 0.21 | 12.80 | 45.63 | **0.02** | **0.03** |
|  | **Avg. number of chr21 genes intersected by duplications** | 0.04 | 0.07 | 0.23 | 0.13 | 0.30 | 0.42 | 0.32 |
| **Registered in**  **DGV** | **Number of deletions** | 61 | 62 | N/A | 36 | 40 | N/A |  |
|  | **Average number of deletions** | 0.35 | 0.34 | 0.59 | 1.50 | 1.33 | 0.75 | 0.80 |
|  | **Average kb covered by deletions** | 26.82 | 26.92 | 0.49 | 13.06 | 21.41 | 0.09 | 0.18 |
|  | **Avg. number of chr21 genes intersected by duplications** | 0.12 | 0.10 | 0.78 | 0.13 | 0.10 | 0.77 | 0.91 |
| **Not registered in DGV** | **Number of deletions** | 5 | 9 | N/A | 0 | 2 | N/A |  |
|  | **Average number of deletions** | 0.03 | 0.05 | 0.26 | 0.00 | 0.07 | 0.30 | 0.28 |
|  | **Average kb covered by deletions** | 69.16 | 95.09 | 0.32 | 0.00 | 132.50 | 0.56 | 0.49 |
|  | **Avg. number of chr21 genes intersected by duplications** | 0.01 | 0.04 | 0.08 | 0.00 | 0.20 | 0.56 | 0.19 |

Table S1. Results of deletion burden tests in PLINK. In Caucasians, we saw no increased burden of average deletion number, average number of bases covered by deletions, or percentage of chromosome 21 genes intersected by deletions. In African Americans, there is a significant increase in bases covered by deletions in DS+NH controls, and this effect is driven primarily by rare deletions.

|  |  | **Caucasian** | | | **African American** | | |  |
| --- | --- | --- | --- | --- | --- | --- | --- | --- |
| **Filtering** | **Burden Test** | **Cases (n = 174)** | **Controls (n = 181)** | **Empirical permuted p-value** | **Cases (n = 24)** | **Controls (n = 30)** | **Empirical permuted p-value** | **Combined p-value** |
| **Chr21** | **Number of duplications** | 31 | 23 |  | 1 | 4 |  |  |
|  | **Average number of duplications** | 0.18 | 0.13 | 0.15 | 0.04 | 0.13 | 0.91 | 0.40 |
|  | **Average kb covered by duplications** | 83.53 | 40.49 | 0.09 | 491.90 | 13.56 | 0.08 | **0.04** |
|  | **Avg. number of chr21 genes intersected by duplications** | 0.22 | 0.10 | 0.07 | 0.33 | 0.03 | 0.44 | 0.13 |
| **Common** | **Number of duplications** | 16 | 17 |  | 0 | 0 |  |  |
|  | **Average number of duplications** | 0.09 | 0.09 | 0.60 | 0.00 | 0.00 | 1.00 | 0.90 |
|  | **Average kb covered by duplications** | 17.67 | 17.41 | 0.43 | 0.00 | 0.00 | 1.00 | 0.79 |
|  | **Avg. number of chr21 genes intersected by duplications** | 0.06 | 0.07 | 0.71 | 0.00 | 0.00 | 1.00 | 0.95 |
| **Rare** | **Number of duplications** | 15 | 6 |  | 1 | 4 |  |  |
|  | **Average number of duplications** | 0.09 | 0.03 | 0.06 | 0.04 | 0.13 | 0.91 | 0.20 |
|  | **Average kb covered by duplications** | 179.30 | 92.39 | 0.13 | 491.90 | 13.56 | 0.08 | 0.06 |
|  | **Avg. number of chr21 genes intersected by duplications** | 0.16 | 0.03 | **0.04** | 0.33 | 0.03 | 0.45 | 0.09 |
| **DGV** | **Number of duplications** | 20 | 20 |  | 0 | 2 |  |  |
|  | **Average number of duplications** | 0.11 | 0.11 | 0.51 | 0.00 | 0.07 | 1.00 | 0.86 |
|  | **Average kb covered by duplications** | 28.50 | 24.63 | 0.34 | 0.00 | 23.62 | 1.00 | 0.70 |
|  | **Avg. number of chr21 genes intersected by duplications** | 0.10 | 0.09 | 0.47 | 0.00 | 0.03 | 1.00 | 0.82 |
| **No DGV** | **Number of duplications** | 11 | 3 |  | 1 | 2 |  |  |
|  | **Average number of duplications** | 0.06 | 0.02 | **0.04** | 0.04 | 0.07 | 0.84 | 0.15 |
|  | **Average kb covered by duplications** | 187.20 | 119.20 | 0.26 | 491.90 | 8.54 | 0.14 | 0.16 |
|  | **Avg. number of chr21 genes intersected by duplications** | 0.11 | 0.01 | **0.03** | 0.33 | 0.00 | 0.44 | 0.08 |

Table S2. Results of duplications burden tests in PLINK. In Caucasians, DS+AVSD cases had significantly more genes intersected by rare duplications than did DS+NH controls, and DS+AVSD cases had a greater number of rare duplications on average.

|  |  | **Caucasian** | | |  |
| --- | --- | --- | --- | --- | --- |
| **Filtering** | **Burden Test** | **DS+AVSD**  **Cases (n = 174)** | **DS+NH**  **Controls (n = 181)** | **Empirical permuted p-value** |  |
| **All** | **Number of CNVs** | 41 | 41 | - |  |
|  | **Average number of CNVs** | 0.24 | 0.23 | 0.62 |  |
|  | **Average kb covered by CNVs** | 50.45 | 46.4 | 0.65 |  |
|  | **Avg. number of chr21 genes intersected by CNVs** | 0.20 | 0.19 | 0.62 |  |
| **Common**  **(≥0.01)** | **Number of CNVs** | 31 | 30 | - |  |
|  | **Average number of CNVs** | 0.18 | 0.17 | 0.67 |  |
|  | **Average kb covered by CNVs** | 40.14 | 38.88 | 058 |  |
|  | **Avg. number of chr21 genes intersected by CNVs** | 0.14 | 0.14 | 0.62 |  |
| **Rare**  **(<0.01)** | **Number of CNVs** | 10 | 11 | - |  |
|  | **Average number of CNVs** | 0.06 | 0.06 | 0.53 |  |
|  | **Average kb covered by CNVs** | 77.31 | 90.28 | 0.38 |  |
|  | **Avg. number of chr21 genes intersected by CNVs** | 0.05 | 0.05 | 0.61 |  |
| **Registered in**  **DGV** | **Number of CNVs** | 37 | 35 | - |  |
|  | **Average number of CNVs** | 0.21 | 0.19 | 0.71 |  |
|  | **Average kb covered by CNVs** | 42.25 | 39.73 | 0.52 |  |
|  | **Avg. number of chr21 genes intersected by CNVs** | 0.17 | 0.16 | 0.67 |  |
| **Not registered in DGV** | **Number of CNVs** | 4 | 6 | - |  |
|  | **Average number of CNVs** | 0.02 | 0.03 | 0.41 |  |
|  | **Average kb covered by CNVs** | 122.3 | 111.6 | 0.58 |  |
|  | **Avg. number of chr21 genes intersected by CNVs** | 0.02 | 0.03 | 0.52 |  |

Table S3. Results of CNVs disrupting exons burden tests. No association was seen with CNVs disrupting genes and returning them to disomy.

|  | **Deletion frequency** | | **Duplication frequency** | |
| --- | --- | --- | --- | --- |
| **Probe Coordinates** | **Cases** | **Controls** | **Cases** | **Controls** |
| CNV1 |  |  |  |  |
| P1.1: chr21:43195435-43195436 | 0.00 | 0.00 | 0.00 | 0.00 |
| P1.2: chr21:43198105-43198106 | 0.00 | 0.00 | 0.00 | 0.02 |
| CNV2 |  |  |  |  |
| P2.1: chr21:43411535-43411536 | 0.00 | 0.00 | 0.00 | 0.02 |
| P2.2: chr21:43413074-43413075 | 0.00 | 0.00 | 0.00 | 0.02 |

Table S4. TaqMan® copy number results of 46 DS+AVSD cases and 46 DS+NH controls. TaqMan® Copy Number assays were performed targeting CNV1 and CNV2, each with two probe-sets. We estimated copy number using CopyCaller™ software. A single control sample had a duplication call at CNV2 by both probes and at CNV1 by one of the two probes. No other CNVs were called by TaqMan.

| **Inclusion criteria** | **Cases** | **Controls** |
| --- | --- | --- |
| Arrays passed Agilent scanner QC of Derivative of Log2 Ratio <0.3 | 236 | 283 |
| Arrays with probe log2 variance <1 SD over mean | 205 | 239 |
| Arrays with probe log2 mean inside 2 SD | 198 | 229 |
| Arrays with variant counts within 5 SD of mean | 198 | 222 |
| Array sample had Affymetrix genotyping data available | 196 | 220 |
| Array not population outlier based on principal component analysis | 196 | 211 |
| **FINAL \| Caucasian** | **174** | **181** |
| **FINAL \| African American** | **24** | **30** |

Table S5. Arrays were required to meet stringent quality control criteria to be eligible for downstream analyses. Analyzed arrays met: 1) Agilent’s recommended Derivative of log_2_ ratio >0.3; 2) intra-array normalized log_2_ ratio variance <one standard deviation above the mean; 3) intra-array normalized log_2_ ratio mean inside two standard deviations; 4) contain fewer deletions or duplications than five standard deviations above the mean; 5) have Affymetrix genotyping data available for principal component analysis (PCA); and 6) not be population outliers identified by PCA.

| **Criteria** | **Deletions** | **Duplications** |
| --- | --- | --- |
| ADM2 called | 486 | 479 |
| GADA called | 2,750 | 2,328 |
| 50% reciprocal overlap | 323 | 428 |
| Variant <1 Mb | 309 | 421 |
| Variant not inside positive control reference deletion | 309 | 67 |
| Variant on q-arm | 307 | 66 |
| Variant not in outlier sample | 226 | 60 |
| Variant not in sample without Affymetrix genotyping data | 224 | 59 |
| Variant not in population outlier identified by Principal component analysis | 215 | 59 |
| **FINAL \| Caucasian** | **137** | **54** |
| **FINAL \| African American** | **78** | **5** |

Table S6. We followed a conservative CNV filtering paradigm to minimize false positives. Analyzed deletions and duplications had to be called by both the ADM2 and GADA algorithms. We removed CNVs over 1 Mb after visualizing log_2_ plots and determining they were likely spurious calls. We removed CNVs within our reference deletion as we did not distinguish absolute copy number of gains or losses and thus could not interpret data over this known deletion in the reference. We removed CNVs on the p-arm or pericentromeric (chr21:0-15,400,000) as these regions are poorly mapped in the reference genome and are gene sparse. Along with those samples, we dropped CNVs in clear outlier samples with more deletions or duplications than five standard deviations over the mean. We dropped CNVs in samples without Affymetrix genotyping data, as well as those CNVs residing in population outliers identified by PCA.

| **CNV coordinates (hg19)** | **Platform** | **Probe Coordinates** |
| --- | --- | --- |
| chr21:43,193,374-43,198,244 | NanoString | chr21:43195101-43195176 |
|  |  | chr21: 43195664-43195743 |
|  |  | chr21:43198103-43198173 |
|  | TaqMan® | chr21:43195435-43195436 |
|  |  | chr21:43198105-43198106 |
| chr21:43,411,411-43,413,231 | NanoString | chr21:43411026-43411115 |
|  |  | chr21:43411401-43411473 |
|  |  | chr21:43412130-43412219 |
|  |  | chr21:43412564-43412653 |
|  |  | chr21:43412999-43413088 |
|  |  | chr21:43413251-43413340 |
|  | TaqMan® | chr21:43411535-43411536 |
|  |  | chr21:43413074-43413075 |

Table S7. NanoString and TaqMan® target probe coordinates for validation of Sailani-associated CNVs.

|  | | | **Quartiles** | | | |
| --- | --- | --- | --- | --- | --- | --- |
|  |  |  | **1** | **2** | **3** | **4** |
| **Deletions** | **Caucasians** | CNV size range | 1792-7767 | 7767-10661 | 10661-48448 | 48448-239099 |
|  |  | **DGV matches (%)** | **27/35 (77)** | **50/50 (100)** | **16/19 (84)** | **32/33 (97)** |
|  | **African Americans** | CNV size range | 2062-4312 | 4312-5372 | 5372-8408 | 8408-260263 |
|  |  | **DGV matches (%)** | **27/27 (100)** | **14/14 (100)** | **17/17 (100)** | **20/20 (100)** |
|  | **All** | CNV size range | 1792-4413 | 4413-10661 | 10661-15500 | 15500-260263 |
|  |  | **DGV matches (%)** | **52/55 (95)** | **89/94 (95)** | **10/12 (83)** | **52/55 (96)** |
| **Duplications** | **Caucasians** | CNV size range | 10981-14435 | 14435-22636 | 22636-59776 | 59776-395106 |
|  |  | **DGV matches (%)** | **13/16 (81)** | **10/11 (91)** | **12/13 (92)** | **6/14 (64)** |
|  | **African Americans** | CNV size range | 6829-10894 | 10894-13517 | 13517-253161 | 253161-491887 |
|  |  | **DGV matches (%)** | **1/2 (50)** | **1/1 (100)** | **1/1 (100)** | **0/1 (0)** |
|  | **All** | CNV size range | 6829-14435 | 14435-20962 | 20962-59776 | 59776-491887 |
|  |  | **DGV matches (%)** | **16/20 (80)** | **9/10 (90)** | **13/14 (93)** | **9/15 (60)** |

**Table S8:** Counts by size quartiles of CNVs discovered in our cohort that have at least 50% reciprocal overlap with the same type of variant (deletion or duplication) registered in The Database of Genomic Variants.
